# Impact of active root zone soil potassium levels on cotton yield and fiber quality under no tillage

**DOI:** 10.3389/fpls.2024.1458367

**Published:** 2024-10-07

**Authors:** Jingjing Shao, Aizhong Liu, Helin Dong, Pengcheng Li, Miao Sun, Weina Feng, Feichao Huo, Cangsong Zheng

**Affiliations:** ^1^ State Key Laboratory of Cotton Biology, Institute of Cotton Research, Chinese Academy of Agricultural Sciences, Anyang, Henan, China; ^2^ Western Research Institute, Chinese Academy of Agricultural Sciences, Changji, China

**Keywords:** cotton, available potassium, yield, quality, spatiotemporal distribution

## Abstract

**Introduction:**

Potassium deficiency significantly hinders cotton growth and development, adversely affecting yield and fiber quality. Applying potassium fertilizer is a common practice to address potassium deficiency in the soil. However, the effectiveness of potassium fertilizer application depends on the appropriate soil potassium levels in cotton fields.

**Methods:**

This study used a randomized block design with six different soil potassium levels and conducted experiments across 18 micro-zones in the field. This study aimed to investigate the response of cotton yield and quality to different soil potassium levels, to try to clarify the suitable soil potassium levels for cotton growth, so as to provide practical and effective help for determining the amount of potash fertilizer in the cotton field.

**Results:**

The results showed that the seedcotton yield was increasing, with the soil potassium level increased under no tillage. There was no significant difference among K4, K5, and K6 on seedcotton yield, which were significantly higher than K1 and K2. As soil potassium levels increased, the proportion of autumn boll and the proportion of outer boll also increased, indicating that higher soil potassium levels support the better growth and development of cotton in the middle and late stages, leading to increased boll sets and higher yields. Additionally, the available potassium content in the 0–40-cm soil layer was significantly correlated with yield and yield parameters but not with fiber quality indices.

**Discussion:**

It is concluded that K4 treatment could provide sufficient potassium to meet the growth and development needs of cotton. Potassium fertilizer application is recommended when the available potassium content in the 0–40-cm soil layer falls below 122.88 mg kg^-1^ in the cotton field.

## 1 Introduction

Potassium plays a crucial role in the growth and development of plants and enhancing crop yields and quality (Oosterhuis et al., 2013; Yang et al., 2016). Cotton, with its unlimited boll-setting habit (Davidonis et al., 2004; Tariq et al., 2018) and unique fiber development mechanisms (Yang et al., 2016), is particularly sensitive to environmental changes, which affect its yield and quality (Chen et al., 2015). Consequently, a substantial amount of potassium is required for optimal yield and quality formation (Adeli and Varco, 2006; Davidonis et al., 2004). Research indicates that cotton is more sensitive to potassium than other crops (Cassman et al., 1989; Cope, 1981). Potassium deficiency can reduce the boll number and boll weight of cotton (Hu et al., 2016b; Read et al., 2006), impacting yield (Li et al., 2012), although it does not affect the lint percentage (Gormus and Yucel, 2002). The adverse effects of soil potassium deficiency on cotton fiber quality are well documented (Oosterhuis et al., 2014; Pettigrew, 2008), and studies have shown that soil potassium deficiency can significantly reduce fiber strength (Girma et al., 2007; Yang et al., 2016), fiber length (Girma et al., 2007), fiber maturity, and micronaire (Pettigrew, 2003, 2008; Pettigrew et al., 2005). It is necessary for cotton growth, yield, and quality formation with sufficient potassium supply.

Applying potassium fertilizer in potassium-deficient soils is a common practice in agricultural production. Moreover, in the pursuit of higher yields, the quantity of potassium fertilizer applied has increased, leading to concerns about its excessive use. Potassium fertilizer primarily comes from potash-bearing salts found in limited national deposits in the world. Potash salt is a unique mineral with no substitutes, and potash resources are classified as “scarce” (Prakash and Verma, 2016). China faces a shortage of soluble potassium mineral resources, and domestic potash fertilizer production cannot meet the consumption demand, necessitating imports for some of its potassium fertilizer needs (Zhang et al., 2016). Consequently, the excessive application of potash fertilizer not only increases costs but also decreases economic benefits. Furthermore, the overuse use of potassium fertilizer impacts potassium cycling within soil–crop systems. Potassium exists in various forms in the soil, maintaining a dynamic equilibrium. Applying potassium fertilizer disrupts this balance, causing some potassium to become fixed and thus unavailable for plant absorption, reducing the effectiveness of potassium fertilizer. It is generally understood that potassium fixation increases with the addition of exogenous potassium (Chen and Mackenzie, 1992; Yadav and Sidhu, 2016). Therefore, excessive potassium fertilizer use fails to improve crop yield and results in low fertilizer use efficiency, leading to a significant waste of potassium resources and environmental pollution (Guo et al., 2010; Pettigrew, 2008). Fertilization practices should ensure that crops receive sufficient potassium while maintaining a reasonable and appropriate application rate in agricultural production.

In soil–crop systems, crop growth response to potassium fertilizer application varies according to the soil’s potassium-supplying capacity (Ladha et al., 2003). Changes in soil-available potassium are a closely related parameter that directly reflects the potassium budget in the soil–crop system (Lu et al., 2017). The soil nutrient level is fundamental to rational fertilization practices. Therefore, fertilization strategies should be tailored to the soil potassium supply level in agricultural production. When developing a potassium fertilizer application program, it is essential to understand the soil potassium supply capacity and the appropriate soil potassium level for cotton. Research has highlighted that transgenic insect-resistant cotton, widely planted in recent years, is even more sensitive to soil potassium deficiency than conventional cotton (Dong et al., 2010; Zhang et al., 2009). Therefore, ensuring an adequate supply of soil potassium is essential for the effective production of cotton. Despite numerous studies on potassium fertilizer application in cotton fields, research on the different effects of cotton yield and quality formation under different soil potassium levels is limited. Our findings will provide a reference for potassium fertilizer application in no-tillage cotton fields and provide practical insights for enhancing cotton yield and fiber quality. Knowledge of the suitable soil potassium content for transgenic cotton, especially under no-till conditions, could improve agricultural efficiency, save costs, and protect the environment. It was hypothesized that the seedcotton yield would not increase with the increase of soil potassium level when the soil potassium content reached a certain concentration, and the spatial and temporal distribution of cotton bolls was associated with soil potassium level in this study in order to determine the suitable soil potassium levels for cotton growth.

## 2 Materials and methods

### 2.1 Experimental site description

**Figure 1:**
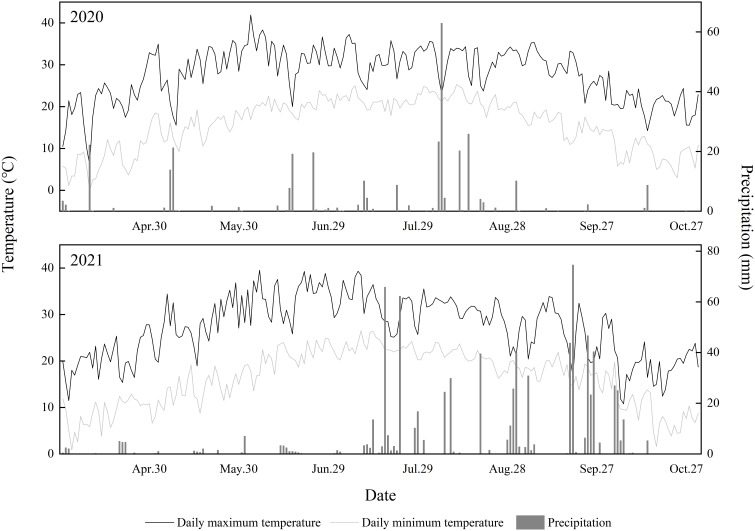
Daily air temperature and precipitation during the cotton growing season from April to October in 2020 and 2021.

The experiment was conducted in 2020 and 2021 at the Experimental Station of the Chinese Academy of Agricultural Sciences’ Institute of Cotton Research, located in Anyang City, Henan Province, China (36°13′ N, 114°35′ W). The site features sandy loam soil. Daily air temperature levels and precipitation during the cotton growing seasons from April to October in 2020 and 2021 are shown in **Figure 1**. Total precipitation was 317.5 mm from April to October 2020 and 785.7 mm during the same period in 2021. No previous crops were planted before cotton sowing in this experimental field, which was planted with a single cotton crop each year. The study adopted a no-tillage design; the experimental field was not ploughed but was only raked.

### 2.2 Experimental design

The field experiment included 18 micro-zones, each measuring 3.6 m in length (north–south) and 4 m in width (east–west), with an area of 14.4 m^2^. The micro-zones were arranged in two rows from east to west, separated by cement structures 10 cm wide. A randomized block experiment was arranged with six treatments, namely K1, K2, K3, K4, K5, and K6, each replicated three times. Before planting in 2020 and 2021, soil nutrients in the experimental micro-zones were assessed. In the 0–40-cm soil layer, the soil contained 11.5 and 10.9 g kg^−1^ organic matter, 0.6 and 0.6 g kg^−1^ total nitrogen, and 12.6 and 9.3 mg kg^−1^ Olsen phosphorus prior to experimental manipulations in 2020 and 2021. The available potassium content for each treatment level is shown in **Table 1**. The no-tillage sowing was on April 25, 2020 and 2021, using upland cotton variety Ji228 as the tested cultivars. Each plot consisted of five rows with south–north orientation, row spacing of 80 cm, and plant spacing of 20 cm. Nitrogen and phosphate fertilizers and field cultivation management practices were consistent across all micro-zones. Nitrogen fertilizer was applied at 225 kg N ha^-1^ (urea, 46% N), with 40% as the basal application before sowing and 60% as topdressing during the early flowering stage. Phosphate fertilizer (triple superphosphate, P_2_O_5_, 42%) was applied at 120 kg P_2_O_5_ ha^-1^ as a single basal fertilizer. All micro-zones were irrigated at the same time and quantity. Other field management practices adhered to high-yielding cotton cultivation standards, ensuring consistency across all micro-zones.

**Table 1:** Soil available potassium level of 0-40cm soil layer in micro area.

Treatment	2021	2022
	(mg kg^-1^)	
K1	100.10c	103.72e
K2	104.18c	109.41de
K3	111.66c	118.69cd
K4	122.88b	127.33bc
K5	131.00ab	134.63ab
K6	137.80a	144.09a
Year	*	
Treatment	**	
Year × Treatment	ns	

Values followed by different letters within the same column are significantly different at the 0.05 probability level. * and ** means the significant differences at 0.05 and 0.01 probability levels, respectively, ns mean no significant difference.

### 2.3 Spatiotemporal distribution of cotton bolls, yield, and yield components

A total of 10 consecutive representative cotton plants with uniform growth were selected in each micro-zone. On July 15, August 15, and September 15 of each year, the number of pre-summer boll, summer boll, and autumn boll, respectively, on these labeled plants were recorded. Prior to harvest, the occurrence and shedding of buds, flowers, and bolls on different fruit branches of the labeled plants were investigated and recorded.

During harvest, all open bolls from the 10 plants in each zone were collected, dried, and weighed to determine the boll weight (seedcotton weight per boll). The bolls were then ginned to calculate the lint percentage. The total seedcotton from each plot was harvested, and the yield was calculated based on the weight of air-dried seedcotton.

### 2.4 Difference of yield

The cotton yield increase was influenced by boll weight and boll number. The proportion of increased yield due to the increase of each factor to the total added value is calculated as follows:


(1) $$\begin{array}{c} \text{Increasing rate because of boll number}\\ =({\text{N}}_{2}-{\text{N}}_{1})\times {\text{W}}_{2}/({\text{N}}_{2}{\text{W}}_{2}-{\text{N}}_{1}{\text{W}}_{1})\times 100\left(\%\right) \end{array}$$

(2) $$\begin{array}{c} \text{Increasing rate because of boll weight}\\ ={\text{N}}_{1}\times ({\text{W}}_{2}-{\text{W}}_{1})/({\text{N}}_{2}{\text{W}}_{2}-{\text{N}}_{1}{\text{W}}_{1})\times 100\left(\%\right) \end{array}$$

N_2_, boll number of the treatment of 2. N_1_, boll number of the treatment of 1. W_2_, boll weight of the treatment of 2. W_1_, boll weight of the treatment of 1.

### 2.5 Fiber quality

The seedcotton was dried and ginned to obtain lint. The fiber quality indicators of cotton were analyzed at the Cotton Quality Supervision, Inspection and Testing Center of the Ministry of Agriculture and Rural Affairs, China.

### 2.6 Statistical analysis

Data were processed using Microsoft Excel 2007, and a data map was drawn using Origin 2018. Statistical analysis was carried out using SPSS 26 version software. The least significant difference test was used for comparing and ranking treatments.

## 3 Results

### 3.1 Yield and yield components

As soil potassium levels increased, the seedcotton yield exhibited an upward trend (**Table 2**). Significant differences among the treatments were observed in seedcotton yield, boll weight, and boll number. Additionally, seedcotton yield and boll weight varied significantly between years. However, no significant interactions were found between year and treatment for boll weight and boll number. The results indicated that there were no significant differences in seedcotton yield among K4, K5, and K6 in 2021, all of which were significantly higher than K1 and K2 in 2020 and 2021 (**Table 2**). The boll weight of K3, K4, K5, and K6 was significantly higher than that of K1, with no significant differences among K3, K4, K5, and K6. Furthermore, the boll number in K4 was significantly higher than in K1 but not significantly different from K3, K5, and K6. The differences in yield across various potassium levels were primarily attributed to variations in boll weight and boll number. Yield analysis further indicated that boll number was the main contributor to yield differences (**Table 3**).

**Table 2:** Effect of different soil potassium level on yield and yield components of cotton.

Year	Treatment	Seedcotton yield	Boll weight per boll	Boll number	Lint percentage
		(kg ha^-1^)	(g)	(no. plant^-1^)	(%)
2020	K1	1212.53d	3.64c	5.67d	40.6a
	K2	1931.74c	3.87bc	9.00c	41.75a
	K3	2705.61bc	5.01ab	10.55bc	41.49a
	K4	3453.66b	5.26a	11.77ab	41.41a
	K5	3341.49b	5.08ab	11.80ab	41.69a
	K6	4649.47a	6.01a	13.93a	40.97a
2021	K1	1661.41c	5.02b	5.98c	37.16a
	K2	2981.12b	5.68a	9.43b	37.72a
	K3	3376.57ab	5.87a	10.27ab	37.91a
	K4	3700.13a	5.92a	11.23ab	37.96a
	K5	3954.02a	5.99a	11.87a	38.12a
	K6	3936.73a	5.91a	12.00a	38.58a
Year		**	**	ns	**
Treatment		**	**	**	ns
Year × Treatment		*	ns	ns	ns

Values followed by different letters within the same column are significantly different at the 0.05 probability level.

* and ** means the significant differences at 0.05 and 0.01 probability levels, respectively, ns mean no significant difference.

**Table 3:** Difference analysis of yield components in yield at different soil K levels.

Treatment	Increasing rate because of boll weight (%)		Increasing rate because of boll number (%)	
	2020	2021	2020	2021
K1				
K2	14.59	26.47	85.41	73.53
K3	45.04	28.98	54.96	71.02
K4	46.22	27.88	53.78	72.12
K5	43.37	28.10	56.63	71.90
K6	52.44	26.17	47.56	73.83

The increasing rate was calculated based on the treatment of K1.

### 3.2 Fruiting branch number and bud and boll falling rate

**Figure 2:**
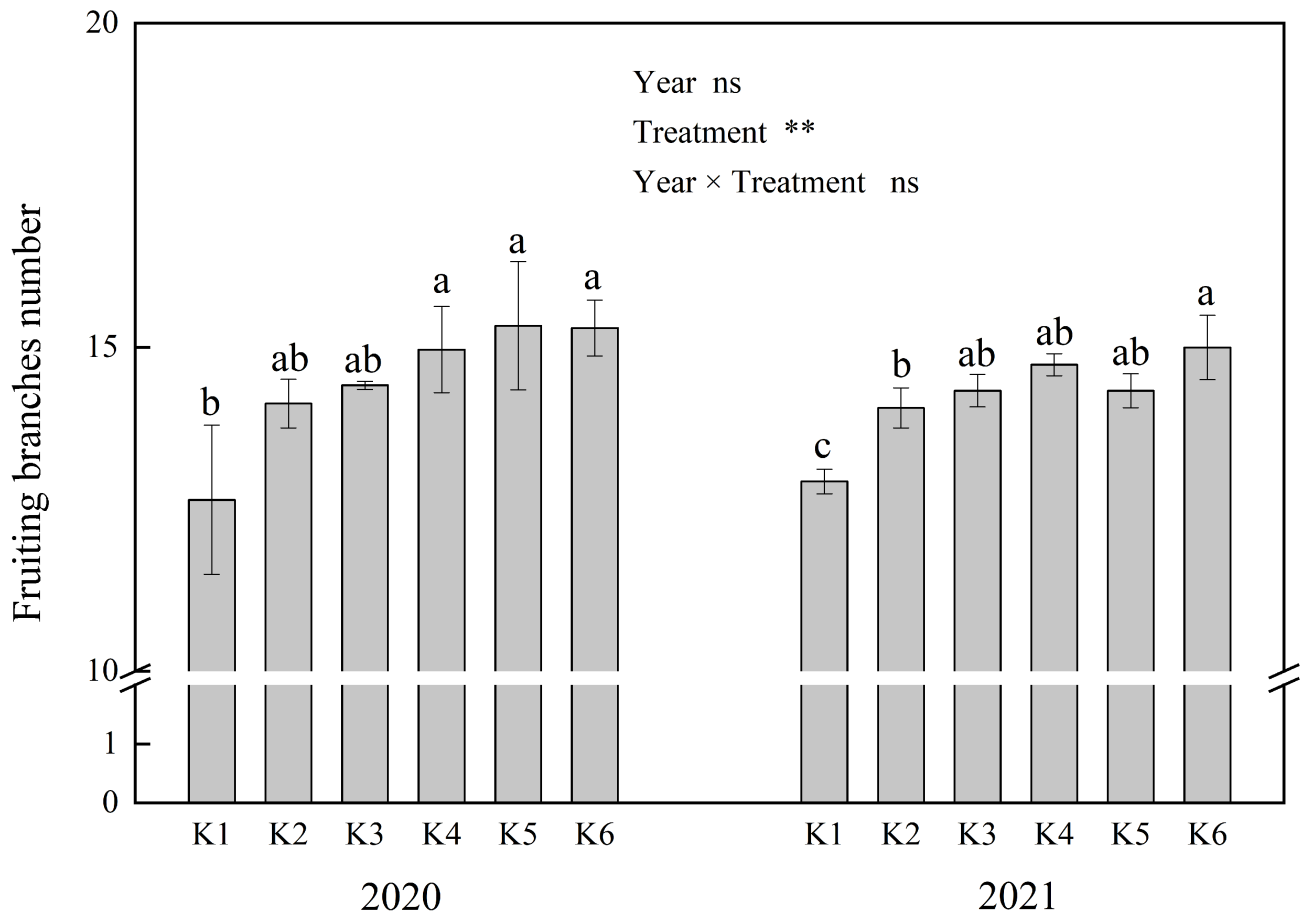
Effects of different soil K levels on fruiting branch number. ** means the significant differences at 0.01 probability level; ns means no significant difference. Values not sharing a common letter within the same year are significantly different at the 0.05 probability level.

The number of fruiting branches per cotton plant did not significantly differ between years but varied significantly among soil potassium levels, with no interaction between year and treatment (**Figure 2**). The number of fruiting branches increased with higher soil potassium levels. The results showed that there were no significant differences in the number of fruiting branches among K3, K4, K5, and K6, all of which were significantly higher than K1 (**Figure 2**).

**Figure 3:**
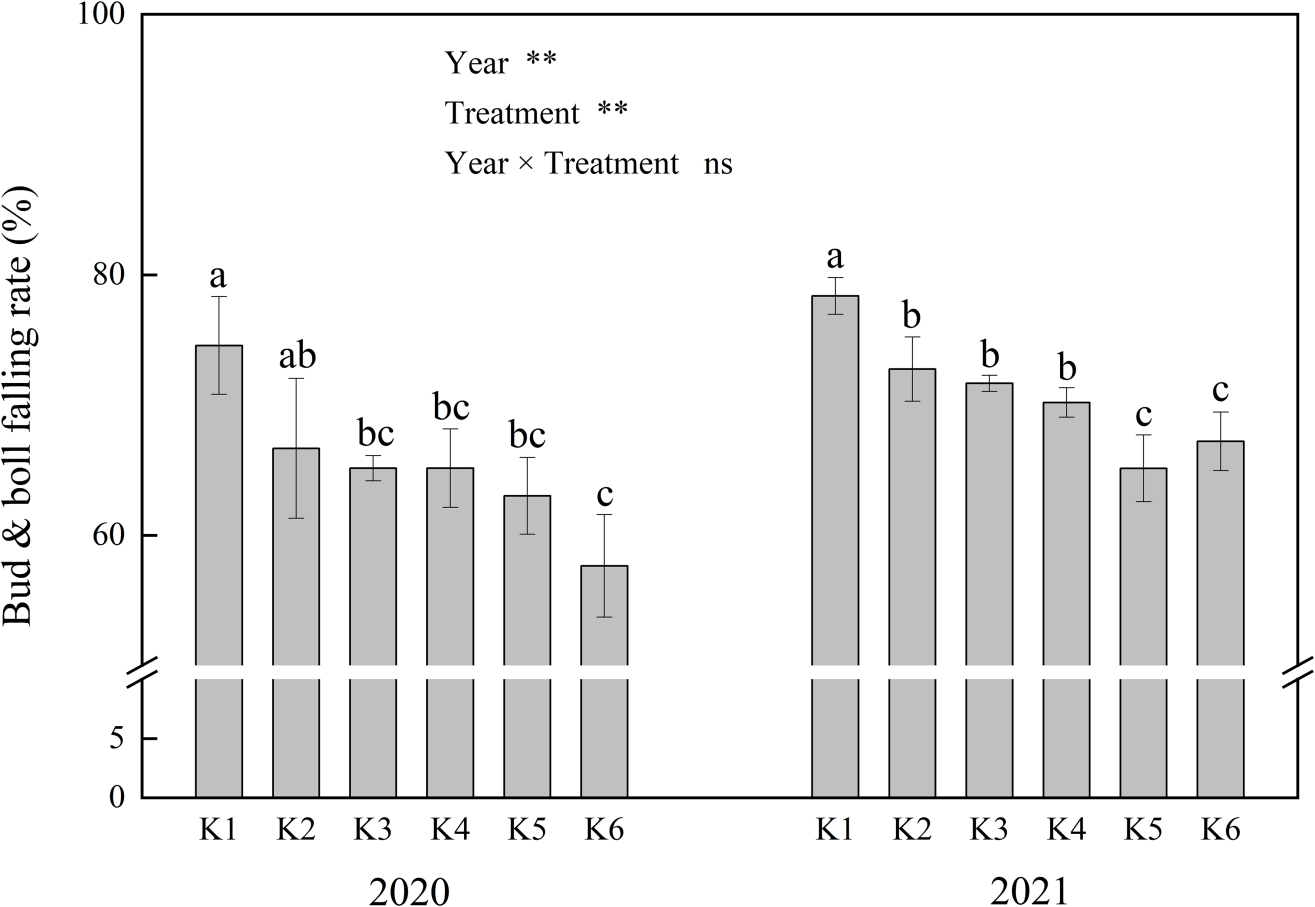
Effects of different soil K levels on bud and bolls falling rate of cotton. ** means the significant differences at 0.01 probability level; ns mean no significant difference. Values not sharing a common letter within the same year are significantly different at the 0.05 probability level.

Significant differences were observed in bud and boll falling rates among soil potassium levels and between years (**Figure 3**). Increasing soil potassium levels significantly reduced the bud and boll falling rates of cotton (**Figure 3**) and increased the boll number (**Table 2**). Compared to K1, the bud and boll falling rates in other treatments were reduced by 7.2%–22.7%, while the number of effective bolls per plant increased by 3.3–8.3. This indicates that the increase in effective boll number was primarily due to the decrease in bud and boll falling rates.

### 3.3 Temporal distribution of cotton bolls

The temporal distribution of cotton bolls revealed that pre-summer bolls accounted for 4%–14% of the total bolls, summer bolls accounted for 51%–77%, and autumn bolls accounted for 14%–39% (**Table 4**). There were no significant differences in pre-summer bolls among different soil potassium levels. Significant differences were observed in summer bolls and autumn bolls between different soil potassium levels but not between years (**Table 4**). Specifically, summer bolls and autumn bolls in K4 were significantly higher than K1 but not significantly different from K2, K3, K5, and K6. These results suggest that higher soil potassium levels increased the number of summer bolls and autumn bolls, providing a basis for yield improvement.

**Table 4:** Temporal distribution of the boll under different soil potassium levels in 2020 and 2021.

Year	Treatment	Time distribution		
		Pre-summer boll	Summer boll	Autumn boll
2020	K1	0.6a	3.7b	0.8c
	K2	0.7a	6.4a	1.6bc
	K3	0.5a	6.8a	2.7bc
	K4	0.9a	7.5a	3.2ab
	K5	0.7a	7.5a	3.4ab
	K6	0.8a	7.7a	5.2a
2021	K1	0.5b	4.6b	0.8c
	K2	1.1ab	6.5a	1.8bc
	K3	1.0ab	6.6a	2.8ab
	K4	1.5a	6.5a	3.2ab
	K5	1.6a	6.1a	4.2a
	K6	1.5a	6.4a	4.1a
Year		**	ns	ns
Treatment		ns	**	**
Year × Treatment		ns	ns	ns

Values followed by different letters within the same column are significantly different at the 0.05 probability level.

* and ** means the significant differences at 0.05 and 0.01 probability levels, respectively, ns mean no significant difference.

### 3.4 Space distribution of cotton bolls

**Figure 4:**
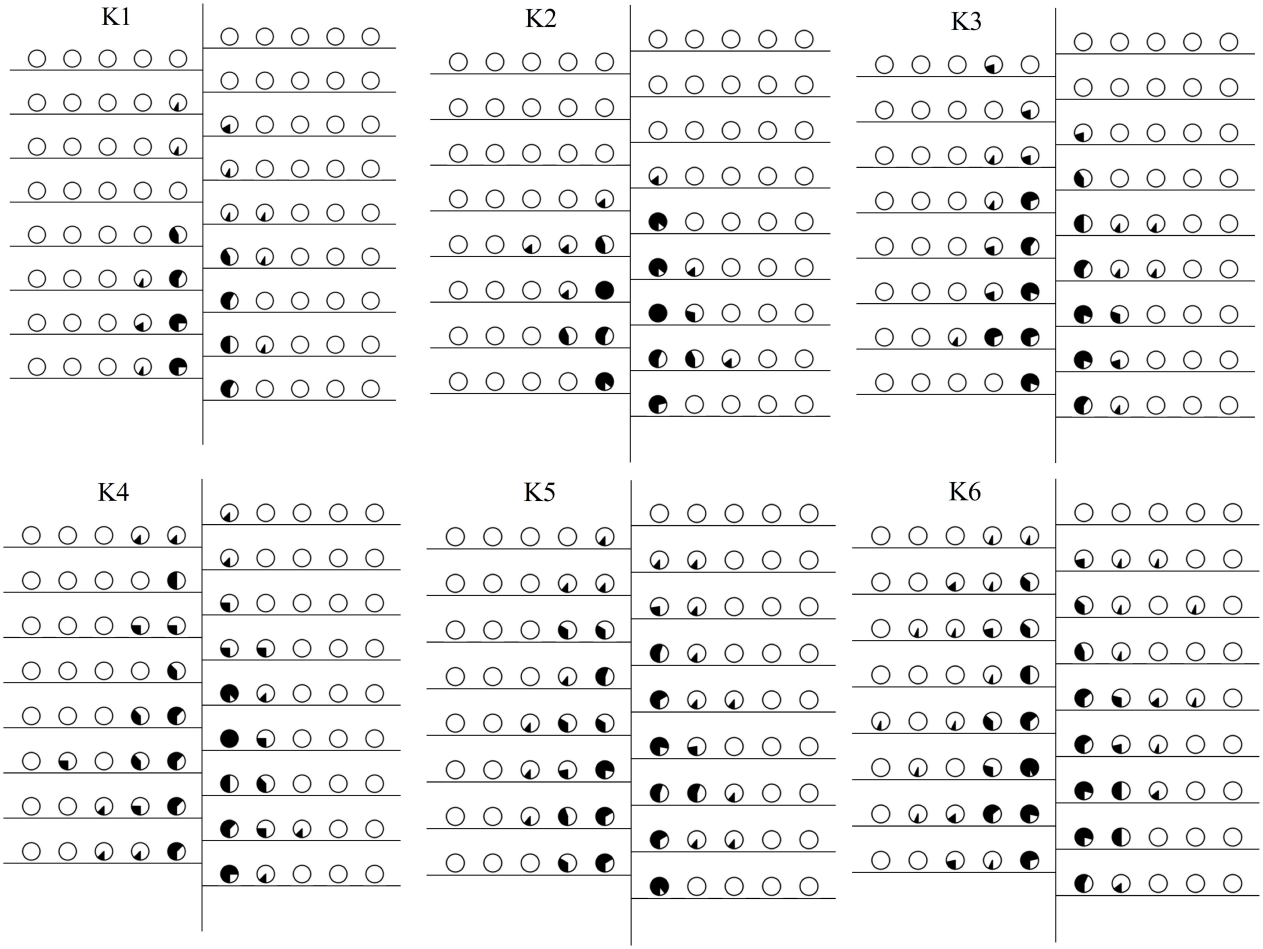
Space distribution of the boll setting rate (%) under different soil potassium levels in 2020. The filled circle stands for boll setting rate and the hollow circle for boll immature rate.

**Figure 5:**
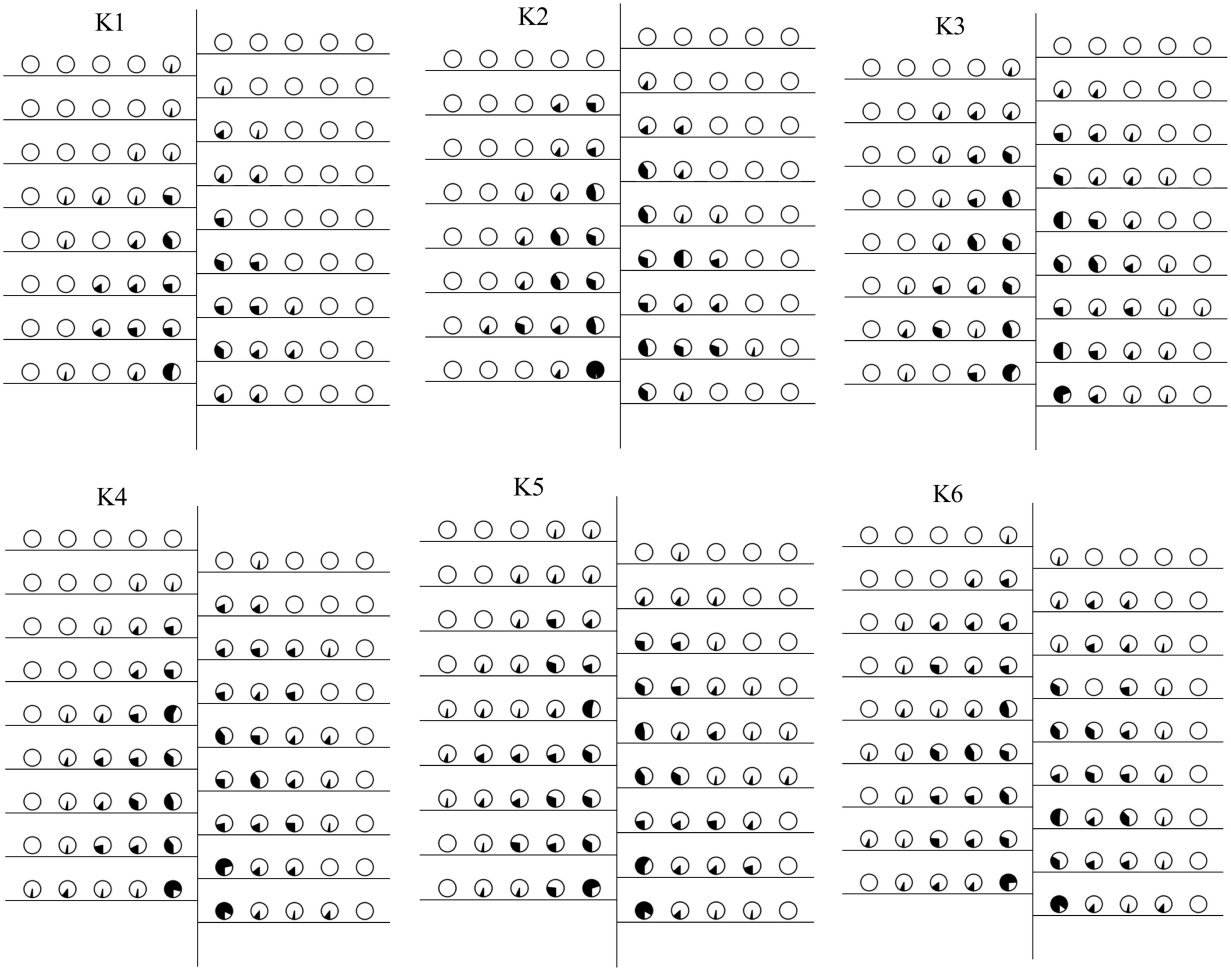
Space distribution of the boll setting rate (%) under different soil potassium levels in 2021. The filled circle stands for boll setting rate and the hollow circle for boll immature rate.

On September 15, we collected mapping information on cotton plants across six soil potassium levels to count the number of boll-setting at various fruiting positions on each fruit branch and to calculate the boll-setting rate (**Figures 4**, **5**). The boll-setting rate decreased from the inside to the outside of the cotton plant, with the rate higher near the main stem than the fruit nodes farther from the main stem. The boll-setting rate also varied among different soil potassium levels. The distribution of bolls from the bottom to the top of the cotton plant (**Figures 4**, **5**) indicated that the number of bolls in the central and upper fruit branches increased with higher soil potassium levels. Specifically, the number of bolls in the lower and central fruit branches of K1 was significantly lower compared to other treatments. In contrast, the number of bolls in the upper fruit branches of K4, K5, and K6 was significantly higher than K1 and K2. The increase in soil potassium levels significantly increased the boll number in each part of the cotton plant, particularly enhancing the proportion of effective bolls in the upper branches.

Regarding the horizontal distribution of boll-setting (**Figures 4**, **5**), the number of bolls increased with higher soil potassium levels in fruit nodes #1–5, especially in the first and second fruit nodes. The boll-setting numbers in the first and second fruit nodes of K1 were significantly lower than those of other treatments. This horizontal distribution analysis suggests that the difference in boll number across soil potassium levels was partially due to increased bolls in the peripheral fruit nodes of cotton plants with higher potassium levels.

### 3.5 Relationship between yield and yield indices

**Figure 6:**
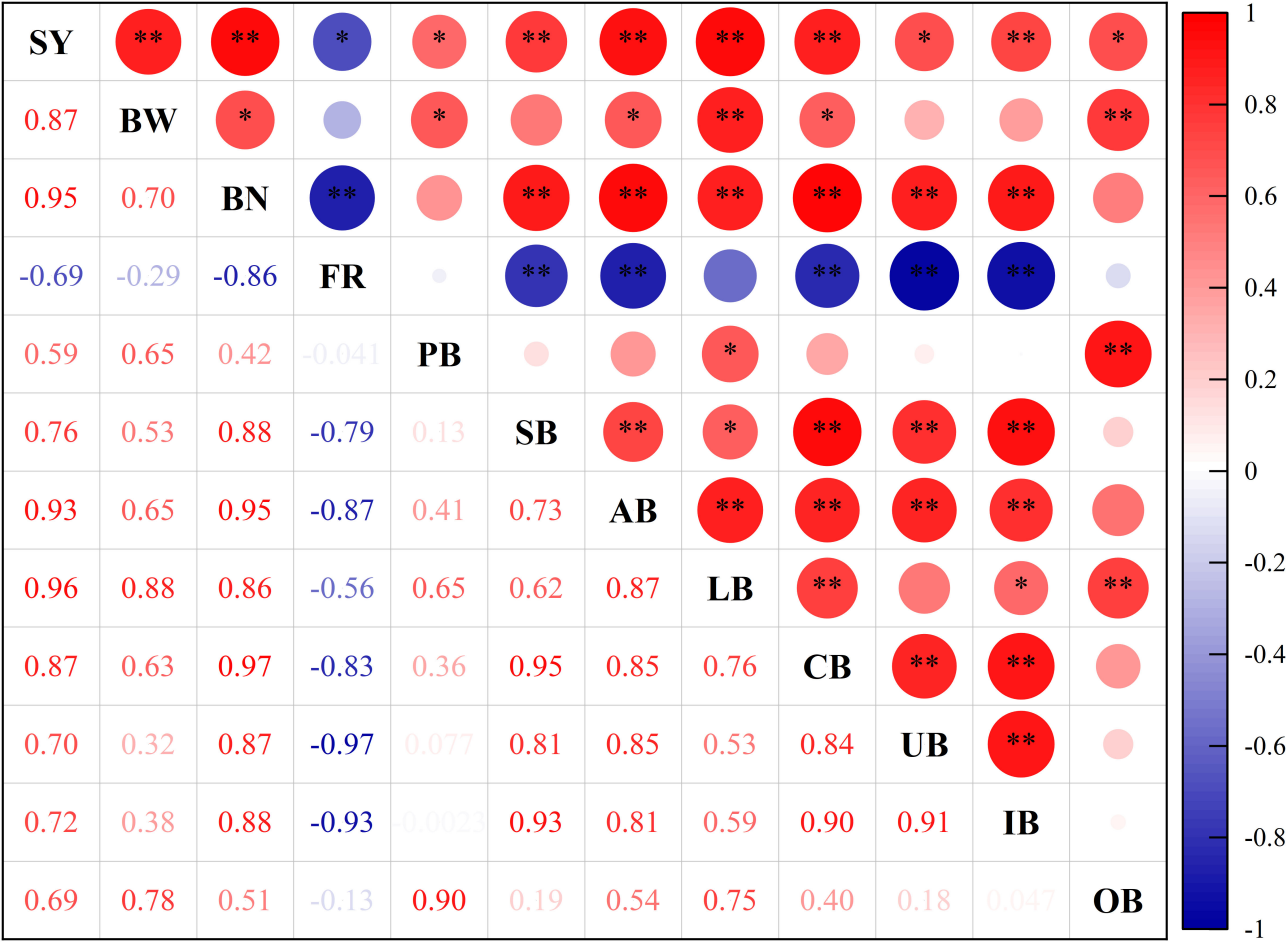
Correlation analysis of yield and yield indices. SY, yield; BW, boll weight; BN, boll number; FR, bud and bolls falling rate; PB, pre-summer boll; SB, summer boll; AB, autumn boll; LB (lower boll), boll number on the first to the fifth fruiting branches; CB (central boll), boll number on the sixth to the 10th fruiting branches; UB (upper boll), boll number on the 11th and above fruiting branches; IB (inner boll), boll number on the first and second fruit nodes; OB (outer boll), boll number on the third and fifth fruit nodes. **Correlation is significant at the 0.01 probability level (two-tailed). *Correlation is significant at the 0.05 probability level (two-tailed).

The correlation matrix analysis of yield and yield indices (**Figure 6**) revealed a marked positive correlation (*P* ≤ 0.01) between boll weight, boll number, summer boll, autumn boll, lower boll, central boll, and inter-boll. Boll number was positively correlated with summer boll, autumn boll, lower boll, central boll, upper boll, and inter-boll (*P* ≤ 0.01). Conversely, the boll falling rate was significantly negatively correlated with boll number, summer boll, autumn boll, central boll, upper boll, and inter-boll (*P* ≤ 0.01).

### 3.6 Fiber quality

Soil potassium levels significantly affected fiber quality parameters. Significant differences were observed between years in upper half mean length, uniformity index, and micronaire. The interactions between year and treatment differed significantly for breaking tenacity, uniformity index, micronaire, and breaking elongation (**Table 5**). In 2020, the fiber quality parameters for K4, K5, and K6 were not different from each other but were significantly higher than K1. In 2021, there were no significant differences among all potassium levels in upper half mean length, breaking tenacity, micronaire, and breaking elongation among K2, K3, K4, K5, and K6 (**Table 5**).

**Table 5:** Effect of different soil potassium level on fiber quality of cotton.

Year	Treatment	Upper half mean length	Breaking tenacity	Uniformity index	Micronaire	Breaking elongation
		(mm)	(cN tex^-1^)	(%)		(%)
2020	K1	27.81c	26.33b	81.29d	3.33c	6.59c
	K2	28.48bc	28.40a	81.69cd	3.63c	6.63b
	K3	28.71abc	29.21a	81.82bcd	4.29b	6.68a
	K4	29.32ab	29.84a	82.56abc	4.64ab	6.70a
	K5	28.84ab	29.40a	82.67ab	4.62ab	6.71a
	K6	29.64a	29.77a	83.28a	4.96a	6.72a
2021	K1	27.87a	28.30a	83.53b	5.60b	6.62b
	K2	28.33a	28.97a	85.00a	6.00a	6.70a
	K3	28.63a	28.83a	85.00a	5.90ab	6.70a
	K4	28.50a	28.77a	84.83ab	5.80ab	6.67a
	K5	28.77a	28.90a	84.53ab	5.87ab	6.70a
	K6	28.17a	28.17a	84.33ab	5.93ab	6.70a
Year		*	ns	**	**	ns
Treatment		*	**	**	**	**
Year × Treatment		ns	**	*	**	*

Values followed by different letters within the same column are significantly different at the 0.05 probability level.

* and ** means the significant differences at 0.05 and 0.01 probability levels, respectively, ns mean no significant difference.

### 3.7 Relationship between soil potassium level and yield and quality indices

**Figure 7:**
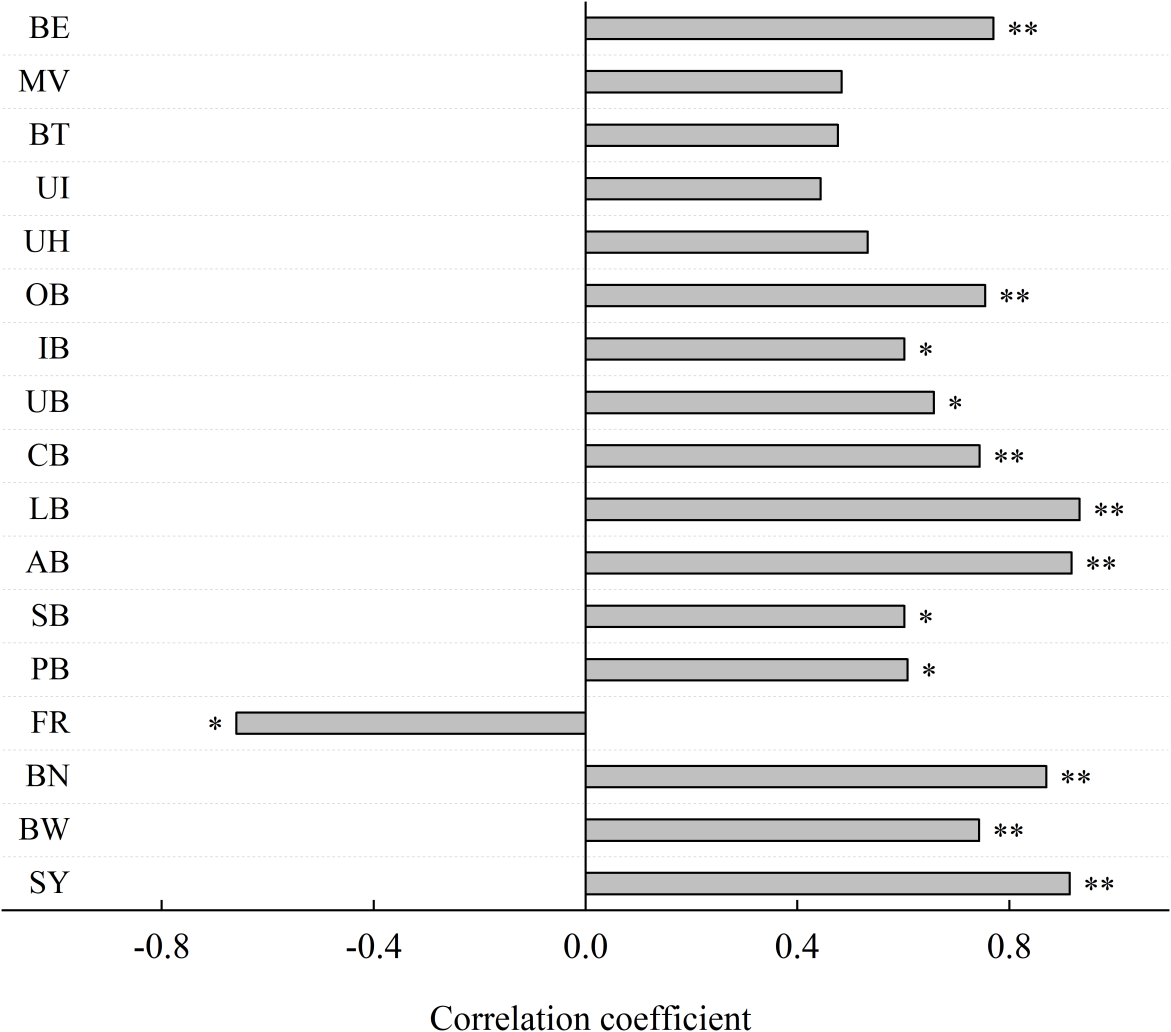
Correlation coefficient between the soil K level and the yield and quality indices. SY, yield; BW, boll weight; BN, boll number; FR, bud and bolls falling rate; PB, pre-summer boll; SB, summer boll; AB, autumn boll; LB (lower boll), boll number on the first to the fifth fruiting branches; CB (central boll), boll number on the sixth to the 10th fruiting branches; UB (upper boll), boll number on the 11th and above fruiting branches; IB (inner boll), boll number on the first and second fruit nodes; OB (outer boll), boll number on the third and fifth fruit nodes; UH, upper half mean length; UI, uniformity index; BT, breaking tenacity; MV, micronaire; BE, breaking elongation. * and ** means the significant differences at 0.05 and 0.01 probability levels, respectively.

The available potassium content in the 0–40-cm soil layer was positively correlated with yield, boll number, boll weight, autumn boll, lower boll, central boll, outer boll, and breaking elongation. The available potassium content also showed a significant positive correlation with the pre-summer boll, summer boll, upper boll, and inner boll. Conversely, a significant negative correlation was observed between the available potassium content and bud and boll falling rate (**Figure 7**). These results suggest that improving the soil potassium supply can enhance the boll setting rate in the later stages of growth, significantly affecting the number of cotton bolls and, ultimately, seedcotton yield. However, the soil-available potassium content had a minimal effect on the fiber quality parameters.

## 4 Discussion

### 4.1 Yield in different soil potassium levels

Adequate potassium supply is essential for achieving a high cotton yield (Gormus, 2002; Oosterhuis et al., 2013). This experiment observed significant differences in cotton yield among different soil potassium levels, with seedcotton yield increasing as soil potassium levels increased. However, no significant differences in seedcotton yield were found between the higher soil potassium levels (K4, K5, and K6). Previous studies have also indicated that further increases in potassium supply do not necessarily lead to higher yields when the soil potassium levels are already high (Shao et al., 2023). Cotton yield is influenced by boll number and boll weight. Potassium deficiency significantly decreases cotton yield (Oosterhuis et al., 2013) by decreasing boll number (Li et al., 2012) and boll weight (Gormus, 2002; Hu et al., 2016a). In this study, a significant positive correlation was found between yield and boll weight and boll number, with the correlation coefficient being higher for boll number. Meanwhile, the yield difference analysis indicated that variations in yield were primarily attributed to differences in boll number across soil potassium levels. Numerous studies have demonstrated that increasing potash fertilizer application can increase the boll number (Hu et al., 2017, 2016; Tariq et al., 2018). However, other studies have reported that potassium fertilizer usage does not affect the boll number (Gormus, 2002) when the soil potassium levels are already high. In this experiment, no significant differences in boll number were observed between the higher soil potassium levels (K4, K5, and K6), suggesting that increasing potassium supply has minimal impact on boll number when the soil potassium levels are sufficient.

The boll number and the bud and boll falling rates determine the effective boll number in cotton. Potassium deficiency can increase the amount of bud and boll shedding (Loka et al., 2018; Zhao et al., 2001), decreasing the boll number. The results showed a significant negative correlation between boll number and the bud and boll falling rate. Increasing soil potassium levels significantly decreased the bud and boll falling rate and increased the boll number. Although the boll number is generally determined based on the total number of bolls collected from the whole plant, few studies have investigated the impact of soil potassium levels on the distribution of boll formation. Boll distribution is an important measure of cotton’s response and adaptation to environmental conditions closely related to yield (Kuai et al., 2015; Pabuayon et al., 2021; Snowden et al., 2013). This study focused on the spatiotemporal distribution of cotton bolls across different soil potassium levels. The temporal distribution of cotton bolls indicated that the proportion of autumn boll increased with increasing soil potassium levels, with a larger correlation coefficient between boll number and autumn boll. Regarding vertical distribution, the lower boll, central boll, and upper boll increased with higher soil potassium levels. Horizontally, the inner boll and outer boll showed an increasing trend with increasing soil potassium levels, although the proportion of the inner boll decreased while the proportion of the outer boll increased. The results suggest that the bolls developing in the later growth stages may not fully mature into open bolls if there is an inadequate supply of potassium. High soil potassium levels can support the growth and development of cotton during the middle and late stages, thereby increasing boll setting in the later stages and maximizing the production potential of cotton plants, ultimately leading to a higher yield.

### 4.2 Fiber quality in different soil potassium levels

Fiber yield is crucial for cotton farmers aiming to maximize profitability, while fiber quality is a key determinant of yarn quality in the textile industry (Bradow and Davidonis, 2000). Therefore, fiber quality significantly impacts the economic value of cotton. Potassium is the most abundant cation in plants (Oosterhuis et al., 2014) and also the most abundant cation in cotton fiber (Almeida et al., 2017). Among fiber quality parameters, fiber length is the most critical factor affecting yarn quality and textile performance (Zhao et al., 2019). Previous experiments have demonstrated that potassium is essential for fiber elongation (Yang et al., 2016; Yu et al., 2023). In 2020, no significant differences were observed in the upper half mean length among K4, K5, and K6, which were significantly higher than K1. Fiber strength is another crucial index of cotton fiber quality, determining yarn strength (Meredith, 2005; Zhao et al., 2020). No significant differences in breaking tenacity were noted among K2, K3, K4, K5, and K6 in 2020 and 2021. Micronaire, a key property in international cotton grading, measures fineness and maturity (Montalvo, 2005). In this experiment, micronaire increased with higher soil potassium levels in 2020. This finding is consistent with previous studies, indicating that micronaire increases with higher potassium application and soil potassium levels (Mullins et al., 1999).

The present study found only a slight and statistically insignificant improvement in fiber quality with increased soil potassium levels. Significant improvements in fiber quality from potassium application observed in previous studies may have been due to lower soil potassium levels (86–92 mg kg^-1^) (Yang et al., 2016). Some studies have reported no improvement in fiber quality with potassium application when the soil potassium level exceeded 150 mg kg^-1^ (Tariq et al., 2018), and no response in micronaire and fiber strength was observed at higher potassium levels (560 and 750 mg kg^-1^ exchangeable potassium) (Gormus, 2002). These results suggest that increased soil potassium level does not enhance fiber quality when the soil potassium level is insufficient.

Fiber quality response to potassium is primarily influenced by genetic composition (Percy et al., 2006), environmental conditions, and management strategies (Subhan et al., 2001). Significant differences in upper half mean length, uniformity index, and micronaire were observed between years, with no significant differences in fiber quality among K2, K3, K4, K5, and K6 in 2021. These variations are likely due to the significantly higher rainfall in 2021 compared to 2020. Previous research has also indicated that water greatly affects fiber quality (Balkcom et al., 2006; Dağdelen et al., 2009; Lokhande and Reddy, 2014). Micronaire is particularly sensitive to water; it increases with adequate irrigation but may decrease with high water supply (Snowden et al., 2013). In this paper, the micronaire in 2021 was significantly higher than in 2020, which was consistent with previous findings.

### 4.3 Cotton yield and quality response to soil potassium level

As a crucial source and sink of nutrients, soil plays a vital role in crop growth. Spatial variation in soil nutrients is common, and understanding this variation is fundamental for effective soil nutrient management and rational fertilization (Zhao et al., 2008). The taproot of annual cotton can extend to a length of 200 cm, while the lateral roots may reach 60–100cm (Rehman and Farooq, 2020). A notable characteristic of the cotton root system is its limited use of surface soil (Brouder and Cassman, 1990). Therefore, it is essential to consider topsoil nutrients and those in deeper soil layers during cotton cultivation. Research has shown that over 73.3% of cotton roots are distributed in the 0–40-cm soil layer (Ping et al., 2012). Thus, at a minimum, soil potassium supply should be evaluated within this 0–40-cm layer.

In this study, increasing soil potassium levels were associated with increases in cotton yield, boll weight, boll number, the number of fruit branches, summer boll, autumn boll, lower boll, central boll, upper boll, inner boll, and outer boll. In contrast, the bud and boll falling rate decreased. Furthermore, the available potassium content in the 0–40-cm soil layer was significantly correlated with yield and various yield parameters. However, the lack of a significant correlation between soil potassium levels and fiber quality indices indicates that increasing potassium does not enhance fiber quality when the soil potassium levels are already sufficient. It is concluded that potassium fertilizer application should be combined with soil potassium levels to maximize cotton yield. According to the results of this study, potassium fertilizer should be considered when the available potassium content in the 0–40-cm soil layer falls below 122.88 mg kg^-1^ in the cotton field.

## 5 Conclusions

In this study, the seedcotton yield increased as the soil potassium level increased under no tillage. The proportion of autumn boll and the proportion of outer boll also increased with the increase of soil potassium level. These findings could ensure the growth and development of cotton in the middle and late stages, thereby increasing the number of boll sets in the later stage and thus increasing the yield under sufficient soil potassium supply. In addition, the soil potassium content of K4 treatment could meet the potassium requirements of cotton growth and development. The application of potassium fertilizer should be considered when the available potassium content in the 0–40-cm soil layer is less than 122.88 mg kg^-1^ (K4) under no tillage.

## Data Availability

The raw data supporting the conclusions of this article will be made available by the authors, without undue reservation.
